# Dedifferentiated liposarcoma lung metastases with different FDG-PET/CT findings

**DOI:** 10.1186/s40792-023-01652-5

**Published:** 2023-05-09

**Authors:** Yoshito Imamura, Satona Tanaka, Akihiko Yoshizawa, Ryo Sakamoto, Hiroshi Date

**Affiliations:** 1 Department of Thoracic Surgery, Kyoto University Hospital, Shogoin-Kawahara-Cho 54, Sakyo-Ku, Kyoto, 606-8507 Japan; 2 Department of Pathology, Kyoto University Hospital, Kyoto, Japan; 3 Department of Diagnostic Imaging, Center for Research on Preemptive Medicine and Lifestyle Related Diseases, Kyoto University Hospital, Kyoto, Japan

**Keywords:** Dedifferentiated liposarcoma, Lung metastasis, FDG-PET/CT

## Abstract

**Background:**

Dedifferentiated liposarcoma (DDLPS) is a rare tumor and generally shows poor prognosis with the lung frequent metastatic site. 18F-fluorodeoxyglucose-positron emission tomography/computed tomography (FDG-PET/CT) is used for staging or metastatic evaluation of this disease. We report a case of bilateral lung metastases of DDLPS showing uncommon imaging on FDG-PET/CT with completely different FDG uptake, which made preoperative diagnosis difficult.

**Case presentation:**

The patient was a male in his 60 s and bilateral lung nodules were noted after proton beam therapy for retroperitoneal DDLPS. FDG-PET/CT showed high FDG uptake in the left S3 15-mm nodule but no uptake in the right S8 10-mm nodule. Thoracoscopic wedge resection for the left nodule was performed, and the pathology revealed metastasis of dedifferentiated liposarcoma. After resection of the left nodule, the right S8 nodule enlarged without FDG uptake. Thoracoscopic right S8 segmentectomy was performed, and metastasis of dedifferentiated liposarcoma was diagnosed. The 2 tumors showed completely different appearances on FDG-PET/CT with similar histopathological findings.

**Conclusions:**

We encountered a case of multiple pulmonary metastases of DDLPS which did not follow the same imaging appearance on FDG-PET/CT. Appropriate timing of surgical resection for pathological diagnosis should be determined carefully.

## Background

Dedifferentiated liposarcoma (DDLPS) is generally associated with a poor prognosis, and the lung is the most frequent metastatic site. 18F-fluorodeoxyglucose-positron emission tomography/computed tomography (FDG-PET/CT) is helpful for staging or metastatic evaluation of this disease, and the dedifferentiated component generally displays high FDG uptake. Here, we report a case of bilateral lung metastases of DDLPS showing uncommon imaging on FDG-PET/CT with completely different FDG uptake, which made preoperative diagnosis difficult.

## Case presentation

**Fig. 1:**
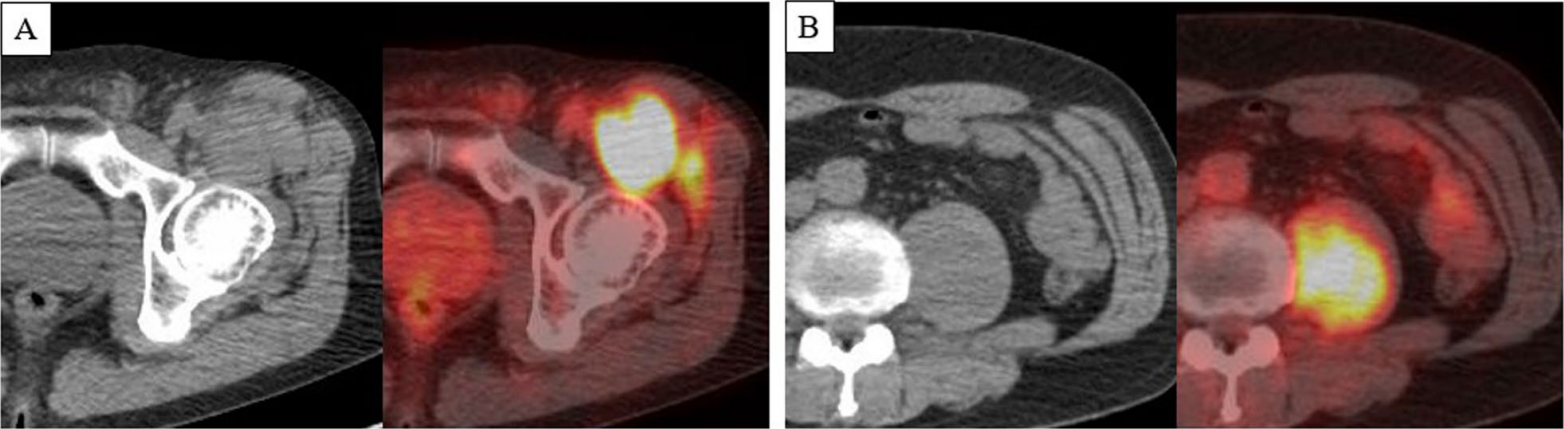
CT and FDG-PET/CT images of retroperitoneal dedifferentiated liposarcoma. FDG-PET/CT showed a SUVmax of 26.1 in the inguinal lesion (**A**) and a SUVmax of 17.8 in the iliopsoas muscle lesion (**B**)

**Fig. 2:**
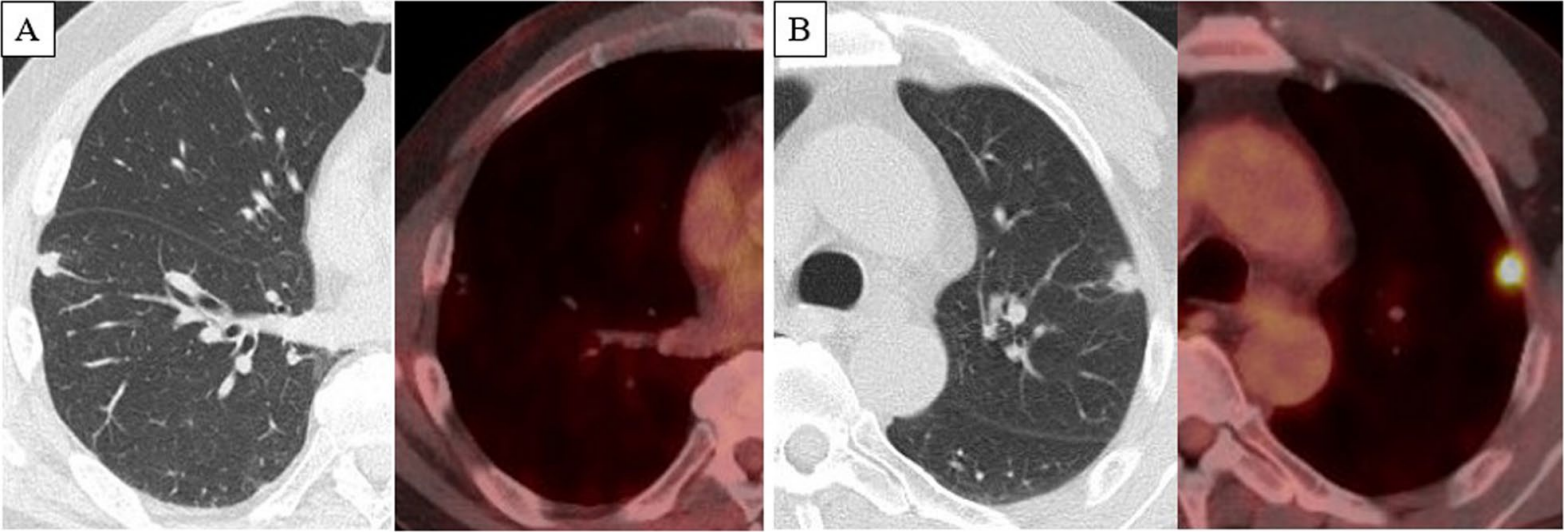
Follow-up CT and FDG-PET/CT at 2 years after treatment. FDG-PET/CT showed a poor FDG uptake in the right S8 10-mm nodule (**A**) but showed FDG uptake (SUVmax 10.7) in the left S3 15-mm nodule (**B**)

**Fig. 3:**
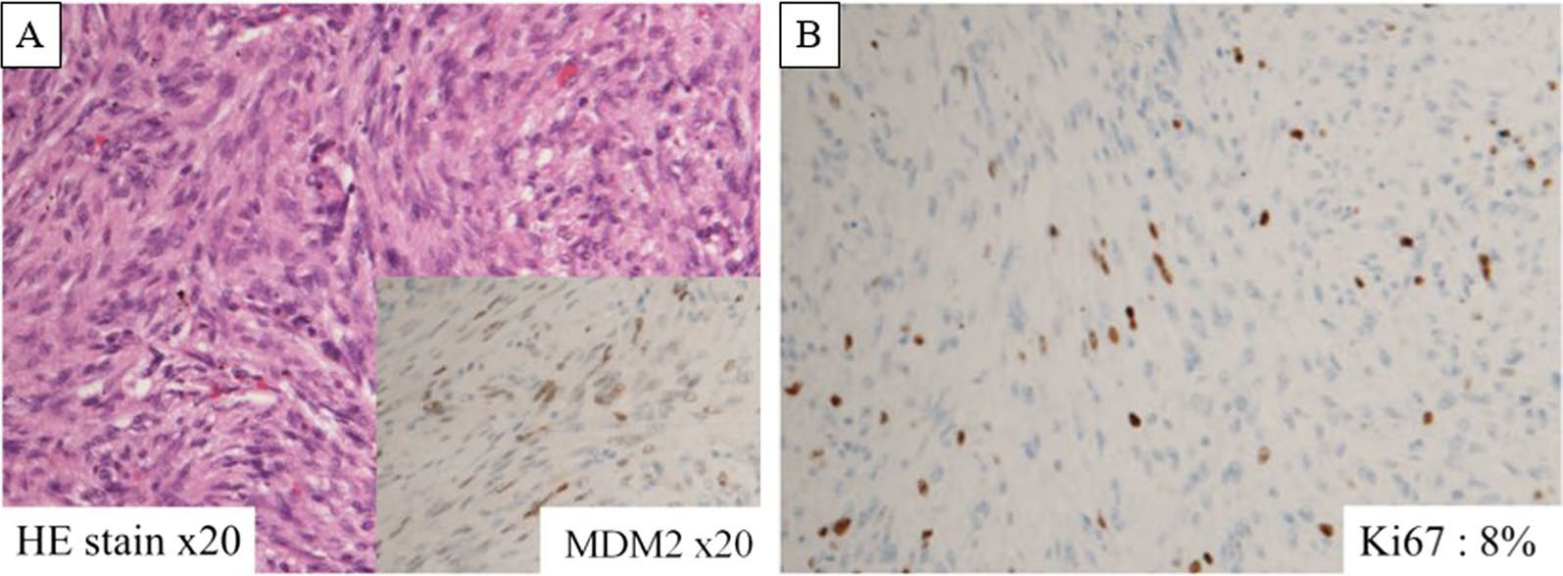
Histopathology and Ki67 labeling index of the left S3 nodule. Histopathology showed a bundle-like proliferation containing spindle cells and positivity for MDM2 (**A**) and the Ki-67 labelling index was 8% (**B**)

**Fig. 4:**
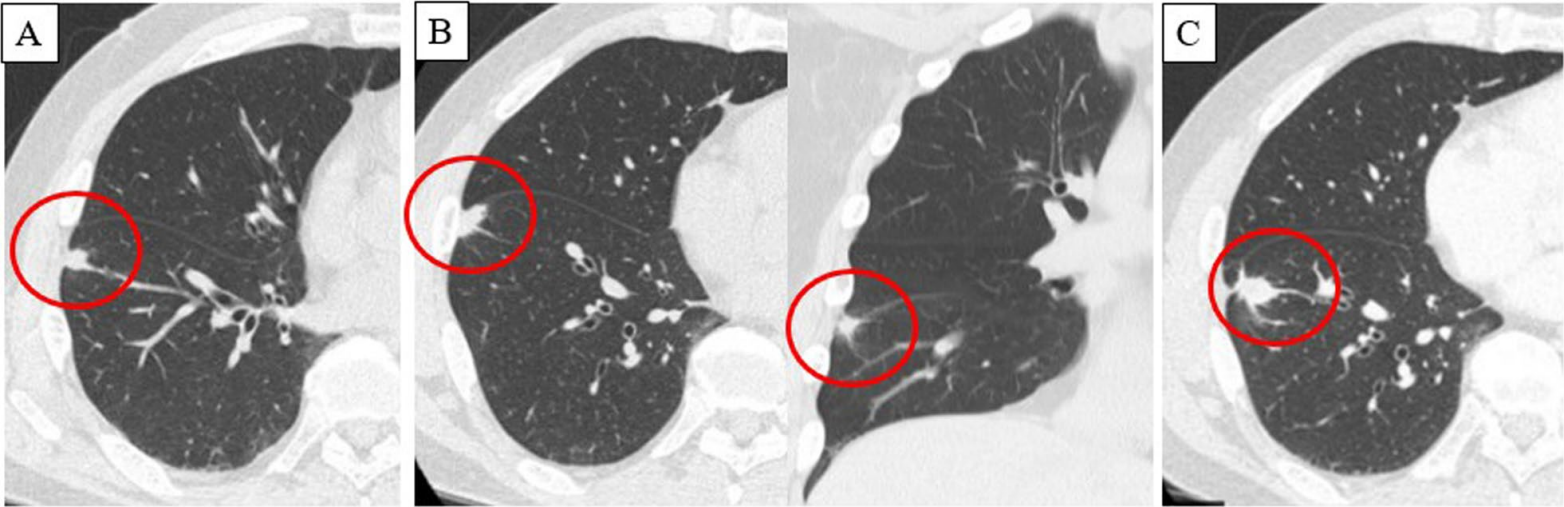
The course of the right S8 nodule on CT. Three months after the first nodal appearance (**A**), the right S8 nodule showed wedge-shaped nodule with linear opacity which was uncommon for a metastatic lung tumor (**B**). One year and three months after the nodule appeared, the size of the right S8 nodule increased to 17 mm (**C**)

**Fig. 5:**
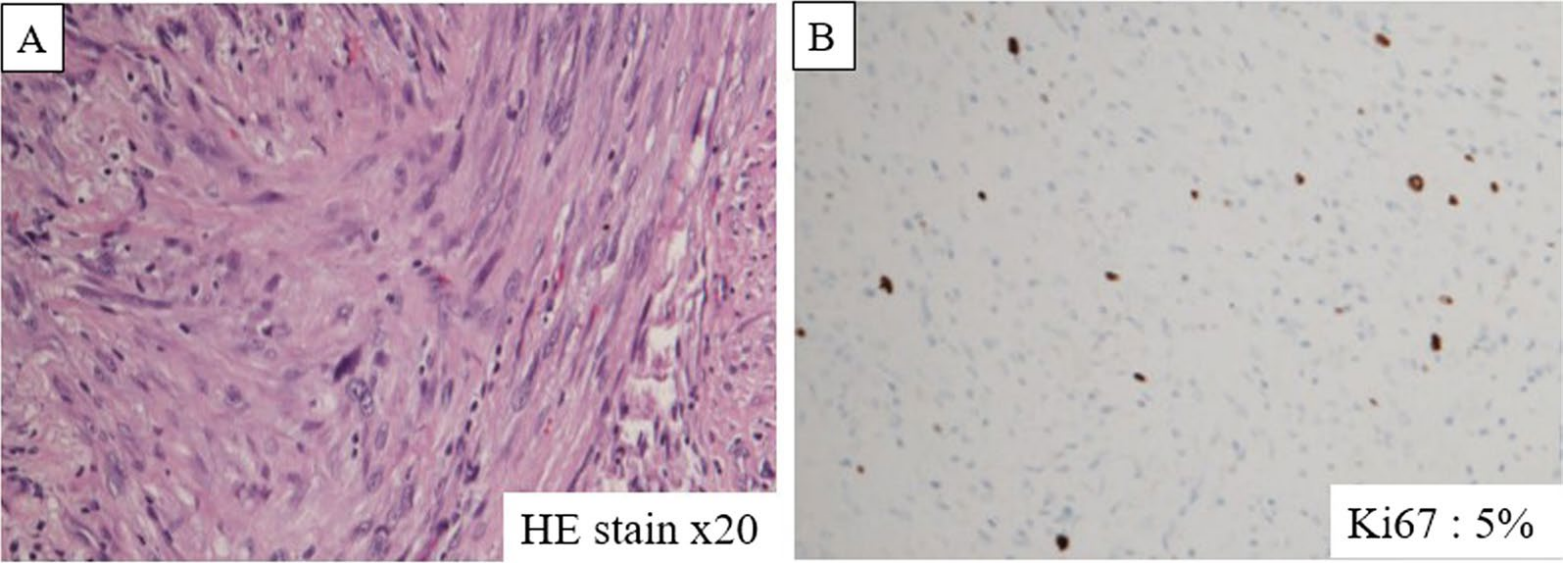
Histopathology and Ki67 labeling index of the right S8 nodule. Histopathology showed a bundle-like proliferation containing spindle cells similar to the findings in the left metastatic tumor (**A**). The Ki-67 labeling index was 5% (**B**)

The patient was a male in his 60 s, presenting with a left inguinal mass. Imaging and biopsy led to a diagnosis of retroperitoneal dedifferentiated liposarcoma (cT2bN0M1 stage IV according to the AJCC/UICC TNM classification). FDG-PET/CT showed a maximum standardized uptake value (SUVmax) of 26.1 in the inguinal lesion and 17.8 in the iliopsoas muscle lesion (Fig. 1). Both lesions were treated with proton beam therapy (70.4 Gy/32 sessions). CT at 1.5 years after initial treatment showed subpleural 10-mm nodules in the left S3 and right S8, respectively. Follow-up CT and FDG-PET/CT at 2 years after treatment showed no enlargement of the right S8 nodule without FDG uptake, and enlargement of the left S3 nodule to 15 mm with high FDG uptake (SUVmax: 10.7) (Fig. 2). The left S3 nodule was suspected to be a metastatic lung tumor, and thoracoscopic wedge resection was performed. Histopathology showed a bundle-like proliferation containing spindle cells and positivity for MDM2; thus, a diagnosis of metastatic DDLPS was made. The Ki-67 labelling index was 8% (Fig. 3). One year and three months after the nodule appeared, the size of the right S8 nodule increased to 17 mm on CT, but FDG-PET/CT still showed poor FDG uptake. In addition, the course of the right S8 nodule on imaging was uncommon for a metastatic lung tumor with wedge-shaped nodule with linear opacity, which made preoperative diagnosis and judging the extent of disease difficult (Fig. 4). As there was no lung nodule except for the right S8 nodule, thoracoscopic right S8 segmentectomy was performed. This nodule was also diagnosed as metastatic DDLPS histopathologically, and there was no obvious difference in the mitotic index, compared with the left S3 nodule. The Ki-67 labeling index was 5% (Fig. 5). Now, at 5 months after the second surgery, the patient is alive without any evidence of recurrence.

## Discussion

Retroperitoneal tumors account for only 0.2% of all tumors. Among them, liposarcomas show the highest frequency of retroperitoneal tumors, at 14.7% [1]. In the WHO classification, liposarcomas are divided into five categories based on their clinicopathological and genetic features: atypical lipomatous tumor/well-differentiated liposarcoma, DDLPS, myxoid liposarcoma, pleomorphic liposarcoma, and mixed-type liposarcoma. DDLPS is a rare and aggressive disease with a six-fold increase in the mortality risk compared with differentiated liposarcomas [2, 3]. It was reported that 30% of dedifferentiated retroperitoneal liposarcomas develop distant metastases, and the lung is the most common distant metastatic site, being associated with decreased survival [4, 5]. Surgical resection is conducted for pathological diagnosis and curative purposes because effective systemic therapy has not been established for metastatic DDLPS.

FDG-PET/CT is useful as an adjunctive method for diagnosing DDLPS. Recurrent tumors of DDLPS tend to show high FDG uptake (SUV Max: 2.3–29.5), and using SUV Max 4 as a cut-off, the diagnostic sensitivity and specificity of DDLPS are 81.8 and 88.9%, respectively [6]. In this case, the left and right metastatic lesions appeared at the same time but showed completely different FDG uptake. DDLPS is heterogeneous, consisting of multiple components and various grades; thus, the heterogeneity of tumors generally explains the difference in FDG uptake. Differentiation was similar in the left and right tumors; therefore, we examined the Ki-67 labelling index because a correlation between FDG uptake and the Ki-67 labelling index was reported in soft tissue sarcomas [7]. The difference in the Ki-67 labelling index between left and right tumors was small, and so we consider that it could not explain the difference in FDG uptake. Although the doubling time of the tumor differed between the left and right nodules (left S3: 105 days, right S8: 207 days), we did not elucidate the reason for this difference in FDG-PET/CT from histopathological findings. The differences in tumor metabolism might underlie and affect glucose metabolism even though differentiation and tumor cell division were similar. Surgeons should note that pulmonary metastasis of DDLPS might appear as uncommon for metastatic nodules, and a lack of FDG uptake does not always rule out metastatic disease of DDLPS.

## Conclusions

We encountered a case of multiple pulmonary metastases of DDLPS which did not follow the same imaging appearance on FDG-PET/CT and preoperative diagnosis was difficult. Appropriate timing of surgical resection for pathological diagnosis should be determined carefully.

## Data Availability

Data sharing is not applicable to this article, as no datasets were generated or analyzed during the study.

## Abbreviations

DDLPS: Dedifferentiated liposarcoma
FDG-PET/CT: 18F-fluorodeoxyglucose-positron emission tomography/computed tomography
SUVmax: Maximum standardized uptake value
